# Children diagnosed with presymptomatic type 1 diabetes through public health screening have milder diabetes at clinical manifestation

**DOI:** 10.1007/s00125-023-05953-0

**Published:** 2023-06-17

**Authors:** Sandra Hummel, Johanna Carl, Nadine Friedl, Christiane Winkler, Kerstin Kick, Joanna Stock, Franziska Reinmüller, Claudia Ramminger, Jennifer Schmidt, Dominik Lwowsky, Sonja Braig, Desiree Dunstheimer, Uwe Ermer, Eva-Maria Gerstl, Leonie Weber, Nicole Nellen-Hellmuth, Susanne Brämswig, Marina Sindichakis, Stefanie Tretter, Anja Lorrmann, Ezio Bonifacio, Anette-G. Ziegler, Peter Achenbach, for the Fr1da Study Group

**Affiliations:** 1 Institute of Diabetes Research, Helmholtz Munich, German Research Center for Environmental Health, Munich, Germany; 2 German Center for Diabetes Research (DZD), Munich, Germany; 3 Forschergruppe Diabetes e.V. at Helmholtz Zentrum München, Munich, Germany; 4 Forschergruppe Diabetes at Klinikum rechts der Isar, School of Medicine, Technical University Munich, Munich, Germany; 5 Nuremberg Hospital South, Nuremberg, Germany; 6 Pediatric Clinic of the Bayreuth Hospital, Bayreuth, Germany; 7 Klinik für Kinder und Jugendliche, Klinikum Augsburg, Augsburg, Germany; 8 St Elisabeth Klinik, Neuburg an der Donau, Germany; 9 Children’s Hospital Dritter Orden, Passau, Germany; 10 Hospital Kempten, Kempten, Germany; 11 MVZ Leopoldina, Würzburg, Germany; 12 RoMed Hospital, Rosenheim, Germany; 13 Hospital Traunstein, Traunstein, Germany; 14 Hospital Weiden, Weiden, Germany; 15 KJF Klinik Josefinum gGmbH, Augsburg, Germany; 16 Center for Regenerative Therapies Dresden, Faculty of Medicine, Technische Universität Dresden, Dresden, Germany; 17 Paul Langerhans Institute Dresden of the Helmholtz Centre Munich at the University Clinic Carl Gustav Carus of TU Dresden and Faculty of Medicine, Dresden, Germany

**Keywords:** Children, Early-stage diagnosis, Islet autoantibodies, Screening, Type 1 diabetes

## Abstract

**Aims/hypothesis:**

We aimed to determine whether disease severity was reduced at onset of clinical (stage 3) type 1 diabetes in children previously diagnosed with presymptomatic type 1 diabetes in a population-based screening programme for islet autoantibodies.

**Methods:**

Clinical data obtained at diagnosis of stage 3 type 1 diabetes were evaluated in 128 children previously diagnosed with presymptomatic early-stage type 1 diabetes between 2015 and 2022 in the Fr1da study and compared with data from 736 children diagnosed with incident type 1 diabetes between 2009 and 2018 at a similar age in the DiMelli study without prior screening.

**Results:**

At the diagnosis of stage 3 type 1 diabetes, children with a prior early-stage diagnosis had lower median HbA_1c_ (51 mmol/mol vs 91 mmol/mol [6.8% vs 10.5%], *p*<0.001), lower median fasting glucose (5.3 mmol/l vs 7.2 mmol/l, *p*<0.05) and higher median fasting C-peptide (0.21 nmol/l vs 0.10 nmol/l, *p*<0.001) compared with children without previous early-stage diagnosis. Fewer participants with prior early-stage diagnosis had ketonuria (22.2% vs 78.4%, *p*<0.001) or required insulin treatment (72.3% vs 98.1%, *p*<0.05) and only 2.5% presented with diabetic ketoacidosis at diagnosis of stage 3 type 1 diabetes. Outcomes in children with a prior early-stage diagnosis were not associated with a family history of type 1 diabetes or diagnosis during the COVID-19 pandemic. A milder clinical presentation was observed in children who participated in education and monitoring after early-stage diagnosis.

**Conclusions/interpretation:**

Diagnosis of presymptomatic type 1 diabetes in children followed by education and monitoring improved clinical presentation at the onset of stage 3 type 1 diabetes.

**Graphical Abstract:**

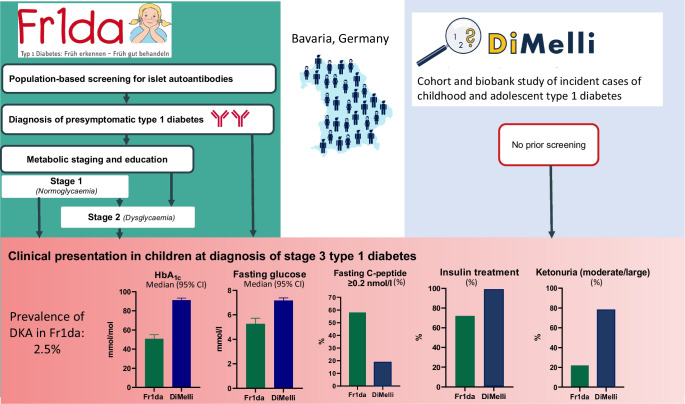

**Supplementary Information:**

The online version contains peer-reviewed but unedited supplementary material available at 10.1007/s00125-023-05953-0.


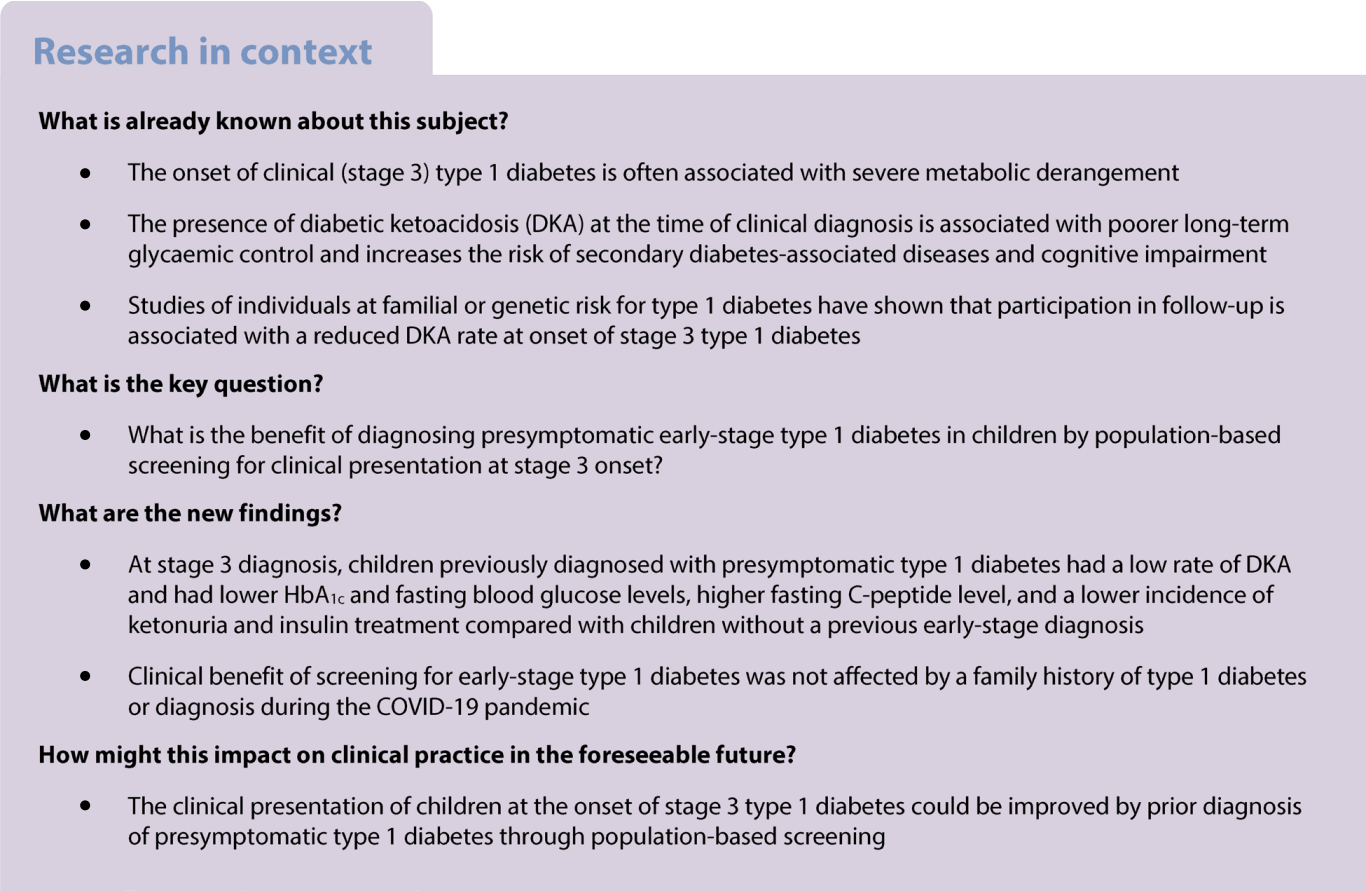


## Introduction

Type 1 diabetes results from an autoimmune destruction of the insulin-producing beta cells [1]. Early presymptomatic stages of the disease are diagnosed by the detection of multiple islet autoantibodies, including autoantibodies against insulin (IAA), GAD (GADA), IA-2 (IA-2A) and ZnT8 (ZnT8A) [2], and are defined as stage 1 (normoglycaemia) or stage 2 (dysglycaemia) type 1 diabetes [3–5]. Several studies in individuals at familial or genetic risk for type 1 diabetes have shown that early-stage diagnosis may have benefits to individuals at clinical onset (stage 3) of type 1 diabetes [6–9]. In particular, diabetic ketoacidosis (DKA), which is observed in 20.3% to 48.4% of individuals at clinical disease onset [10], is reported in less than 10% of individuals who have a prior diagnosis of stage 1 or stage 2 type 1 diabetes [7–9, 11]. The presence of DKA at the time of clinical diagnosis is associated with poorer long-term glycaemic control and increases the risk of diabetic secondary diseases and cognitive impairment [12–15]. In addition, the acute and severe onset of the child’s disease causes psychological distress in parents and children [16].

In 2015, we initiated the Fr1da study in Bavaria, Germany, designed as a model project to evaluate population-based screening for multiple islet autoantibodies for early detection of type 1 diabetes in children [17, 18]. The study is conducted in collaboration with primary care paediatricians, and over 165,000 children have participated to date. The main purpose of the screening is to diagnose presymptomatic type 1 diabetes so that affected children and their families can be educated and monitored, and so that patients can begin insulin therapy early enough to prevent serious metabolic derangements at the onset of clinical disease. The aim of the current study was to determine whether disease severity at the onset of stage 3 type 1 diabetes is reduced in children previously diagnosed with presymptomatic type 1 diabetes in the Fr1da study. We compared the clinical presentation of Fr1da children at stage 3 diagnosis with that of children participating in the DiMelli study, a paediatric diabetes registry that enrolled children with incident stage 3 type 1 diabetes diagnosed through the same clinical centres in Bavaria as in the Fr1da study but without prior screening for islet autoantibodies.

## Methods

### Study design

The present study was conducted in children diagnosed with presymptomatic type 1 diabetes in the Fr1da public health screening programme [17–19] who subsequently developed stage 3 type 1 diabetes. Between February 2015 and 6 December 2022, 169,446 children participated in screening for islet autoantibodies offered by primary care paediatricians in the context of medical check-ups in Bavaria. Children without known diabetes aged 1.75–5.99 years (February 2015 to March 2019) or 1.75–10.99 years (since April 2019) were eligible for screening. Presymptomatic type 1 diabetes was diagnosed when multiple islet autoantibodies tested positive in an initial capillary blood screening sample and in a second venous blood confirmatory sample. Screening samples were tested for GADA, IA-2A and ZnT8A using the 3Screen ELISA (RSR, Cardiff, UK) [20, 21]. Samples above a threshold of 25 U in the ELISA (98th percentile of child samples) and confirmatory samples were tested for GADA, IA-2A, ZnT8A and IAA using radio-binding assays as previously described [19, 22–24].

Families of children diagnosed with presymptomatic type 1 diabetes were invited to participate in metabolic staging and an educational programme at a clinical referral centre near their home, where they received training in urine and blood glucose monitoring, and information on normal and pathological blood glucose levels and on symptoms of hyperglycaemia and DKA [17]. Families were provided with a guidebook (‘Fr1da book’) specifically designed for children with presymptomatic type 1 diabetes and assigned a contact person from the local diabetes centre to whom they could turn at any time if they had questions. Metabolic staging into stage 1 (normoglycaemia), stage 2 (dysglycaemia) or stage 3 (hyperglycaemia) of type 1 diabetes was based on the OGTT and HbA_1c_ levels, according to the 2015 JDRF, Endocrine Society and ADA consensus criteria, as previously described [5]. Dependent on the staging result, children were monitored at intervals of 3 months (stage 2) to 6 months (stage 1) for development of stage 3 type 1 diabetes, which was diagnosed based on ADA criteria [3]. Clinical referral centres were provided with recommendations regarding the timing of initiation of insulin therapy in children with presymptomatic type 1 diabetes (HbA_1c_ >47.5 mmol/mol [>6.5%] or fasting glucose >8.0 mmol/l [>145 mg/dl] or postprandial blood glucose >11.1 mmol/l [>200 mg/dl] [25]).

Families of children who declined staging and/or monitoring were contacted by telephone and asked if the child had developed stage 3 type 1 diabetes. All children diagnosed with stage 3 type 1 diabetes by 6 December 2022 were included in the current analysis. The study was approved by the institutional review board at the Technical University of Munich. Written, informed consent was obtained from the children’s parents or legal guardians.

### Clinical outcome variables at diagnosis of stage 3 type 1 diabetes

At diagnosis of stage 3 type 1 diabetes, local physicians at the clinical referral centre, hospital or primary care centre were asked to complete a structured questionnaire including information on HbA_1c_, fasting glucose, insulin treatment (yes/no), ketonuria (moderate or large), venous pH and/or serum bicarbonate, clinical symptoms (yes/no), hospitalisation (yes/no) and duration of hospitalisation (days), and whether the child was admitted to the intensive care unit (ICU) (yes/no).

DKA was defined according to International Society for Pediatric and Adolescent Diabetes (ISPAD) Clinical Practice Consensus Guidelines 2022 for the diagnosis of DKA [26], including the following biochemical criteria: hyperglycaemia (blood glucose >11 mmol/l [200 mg/dl]), venous pH <7.3 or serum bicarbonate <18 mmol/l, and ketonaemia (blood ß-hydroxybutyrate ≥3 mmol/l) or moderate or large ketonuria. For children with incomplete information on these variables, information on DKA (yes/no) was retrieved from medical records provided by physicians.

Additionally, the questionnaire included information on weight and height, which was used to calculate BMI and convert it into sex- and age-adjusted BMI-SD scores (SDSs) using national reference data [27]. At the time of stage 3 type 1 diabetes diagnosis, a fasting aprotinin-stabilised EDTA venous blood sample was requested to measure C-peptide levels on an automated immunoassay analyser (AIA-360, Tosoh, San Francisco, CA, USA).

Genotyping for 46 SNPs, which have been used to calculate a genetic risk score (GRS) as described previously [28], was performed if consent for ancillary research was provided.

### Demographic variables

Demographic data of the participating children (date of birth, sex, date of blood collection, first-degree family history of type 1 diabetes) were collected using a questionnaire at the time of screening for islet autoantibodies by primary care paediatricians.

### Comparison cohort

The clinical presentation at diagnosis of stage 3 type 1 diabetes in children in the Fr1da study was compared with that of children diagnosed with incident type 1 diabetes without prior screening for islet autoantibodies from the DiMelli study. This is a cohort and biobank study in Bavaria that enrolled incident cases of childhood and adolescent diabetes between 2009 and 2018. The design of the DiMelli study has been described previously [29]. Children who participated in both the Fr1da and DiMelli studies were assigned to the Fr1da cohort for our analysis and were not considered in the DiMelli cohort. At the time of enrolment into the DiMelli study, a fasting blood sample was requested and a structured questionnaire was completed by the local hospital or primary care centre physician, which contained information on patient status, including sex, the date of diagnosis of diabetes, first-degree family history and current glucose-lowering medications. The blood sample was used to determine C-peptide, HbA_1c_, islet autoantibodies, HLA genotyping and SNP analysis [30]. The current analysis included 736 children who were diagnosed before the age of 10.99 years and tested positive for ≥1 islet autoantibody within a median of 1.3 weeks (IQR 0.9–1.9 weeks) of diagnosis. Twenty-nine of the 736 children were enrolled >2 months after diagnosis. The study was approved by the ethical committee of Bavaria, Germany (Bayerische Landesaerztekammer, no. 08043).

### Statistical analysis

As most of the continuous variables were not symmetrically distributed, descriptive values were expressed as median and IQR for continuous variables and as total number (*n*) and percentage (%) for frequencies. For the comparison of characteristics between Fr1da and DiMelli study cohorts and the comparison of clinical outcome variables between Fr1da subgroups, Fisher’s exact test or non-parametric Mann–Whitney *U* test was applied. For the comparison of clinical outcome variables between Fr1da and DiMelli study participants, multivariate linear or logistic regression analysis was applied, adjusting for sex, age and calendar year at diagnosis of stage 3 type 1 diabetes and having a first-degree relative with type 1 diabetes. Results were expressed as estimated difference in means or OR and 95% CI from the multivariate analysis. Numbers for each specific outcome measure are included in the results. Complete-case sensitivity analyses for the comparison of clinical outcome variables between Fr1da and DiMelli study cohorts were performed including only children from both cohorts who had complete information on the following variables: age at stage 3 diagnosis, sex, family history of type 1 diabetes, HbA_1c_, fasting glucose, fasting C-peptide, insulin treatment and ketonuria. All statistical analyses were performed with IBM SPSS Statistics version 28 (IBM, Armonk, NY, USA). Figures were created with GraphPad Prism version 9.5.0 (Graphstats Technologies, USA). The significance level was set at 5% (two-tailed) for all analyses.

## Results

**Fig. 1:**
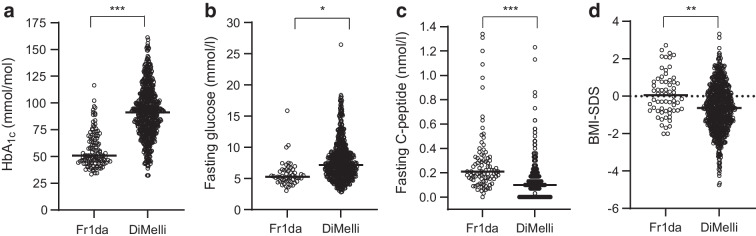
HbA_1c_ (**a**), fasting glucose (**b**), fasting C-peptide (**c**) and BMI-SDS (**d**) at diagnosis of stage 3 type 1 diabetes are shown for children previously diagnosed with presymptomatic type 1 diabetes in the Fr1da study compared with children diagnosed with incident type 1 diabetes in the DiMelli study who had not participated in prior screening. *p<0.05, **p<0.01, ***p<0.001

Of 169,446 children enrolled in the Fr1da study, 473 (0.3%) children tested positive for multiple islet autoantibodies in the screening and confirmatory samples and were diagnosed with presymptomatic type 1 diabetes; they were offered participation in metabolic staging, diabetes education and follow-up at clinical referral centres (electronic supplementary material [ESM] Fig. 1). Of these 473 children, metabolic staging and education were refused or pending for 103 children (22%). A total of 128 of the 473 children (59 girls) progressed to stage 3 type 1 diabetes within a median of 2.3 years (IQR 1.1–4.3 years). Among the children with valid data, the median pH at diagnosis of stage 3 type 1 diabetes was 7.40 (IQR 7.38–7.42) and three (2.5%) of 118 children had a laboratory diagnosis of DKA (Table 1). Clinical symptoms were reported in 53 (43.8%) of 121 children, and children were hospitalised for a median of 8 days (IQR 0–11 days). Two (1.9%) of 108 children were admitted to the ICU.

**Table 1:** Characteristics of the study cohort

Characteristic	Fr1da		DiMelli		*p* value
	*n*	Median (IQR) or *n* (%)	*n*	Median (IQR) or *n* (%)	
Age at diagnosis of stage 3 T1D (years)	128	6.7 (5.0–9.1)	736	7.2 (4.5–9.2)	0.8
Sex	128		736		
Female		59 (46.1)		362 (49.2)	0.6
Male		69 (53.9)		374 (50.8)	
First-degree relative with T1D	128	18 (14.1)	732	49 (6.7)	<0.01
HLA genotype	108		594		0.8
*DR3/4-DQ8*		29 (26.9)		142 (23.9)	
*DR4-DQ8/DR4-DQ8*		7 (6.5)		40 (6.7)	
*DR3/DR3*		4 (3.7)		35 (5.9)	
Other		68 (62.9)		377 (63.5)	
GRS	99	13.1 (11.8–14.2)	587	13.0 (11.9–13.9)	0.7
DKA	118	3 (2.5)			
pH	68	7.40 (7.38–7.42)			
Symptoms	121	53 (43.8)			
Hospitalisation	121	90 (74.4)			
Days	117	8.0 (0–11.0)			
ICU (yes)	108	2 (1.9)			

T1D, type 1 diabetes

The 128 children of the Fr1da study who developed stage 3 type 1 diabetes with a prior early-stage diagnosis were compared with the 736 children with incident type 1 diabetes of the DiMelli study with respect to their clinical presentation at stage 3 diagnosis (Table 1). The children from the two studies did not differ significantly in age at diagnosis of stage 3 type 1 diabetes (median [IQR], 6.7 years [5.0–9.1] vs 7.2 years [4.5–9.2]; *p*=0.8), sex (girls, 46.1% vs 49.2%; *p*=0.6), frequency of type 1 diabetes risk genes (HLA risk genotypes, 37.1% vs 36.5%; *p*=0.8) or GRS (median [IQR], 13.1 [11.8–14.2] vs 13.0 [11.9–13.9]; *p*=0.7). Children from the Fr1da study more frequently had a first-degree family history of type 1 diabetes (14.1% vs 6.7% in DiMelli; *p*<0.01).

### Clinical presentation at diagnosis of stage 3 type 1 diabetes

There were substantial differences in metabolic variables at onset of stage 3 type 1 diabetes between children with and without a prior early-stage diagnosis (Table 2). Children in the Fr1da study had lower HbA_1c_ levels (median [IQR], 51 mmol/mol [45–67] vs 91 mmol/mol [75–107]; 6.8% [6.2–8.3] vs 10.5% [9.0–11.9]; *p*<0.001), lower fasting glucose levels (median [IQR], 5.3 mmol/l [4.6–6.4] vs 7.2 mmol/l [5.7–9.1]; 95 mg/dl [82–116] vs 129 mg/dl [103–164]; *p*<0.05) and higher fasting C-peptide levels (median [IQR], 0.21 nmol/l [0.15–0.33] vs 0.10 nmol/l [0–0.17]; *p*<0.001) at diagnosis of stage 3 type 1 diabetes (Fig. 1a–d). The proportion of children with fasting C-peptide levels ≥0.2 nmol/l was higher in the Fr1da study (58.4%) as compared with DiMelli (18.8%; *p*<0.001). This was accompanied by a decreased frequency of participants requiring insulin treatment at the diagnosis of stage 3 type 1 diabetes (72.3% vs 98.1% in children from the DiMelli study; *p*<0.05) and a decreased frequency of participants with moderate or large ketonuria (22.2% vs 78.4%; *p*<0.001) in children with a prior early-stage diagnosis (Table 2). Children in the DiMelli study had lower BMI-SDS (median [IQR], −0.63 [−1.47 to 0.23] vs 0.05 [−0.77 to 0.77]; *p*<0.01).

**Table 2:** Clinical presentation at diagnosis of stage 3 type 1 diabetes in children previously diagnosed with presymptomatic type 1 diabetes in the Fr1da study compared with children diagnosed with incident type 1 diabetes in the DiMelli study who had not participated in prior screening

Variable	Fr1da		DiMelli		Multivariate regression analysis			
	*n*	Median (IQR) or *n* (%)	*n*	Median (IQR) or *n* (%)	*n*	β estimate (95% CI)	OR (95% CI)	Adjusted *p* value^a^
HbA_1c_ (mmol/mol)	120	51 (45–67)	667	91 (75–107)	784	−40.9 (−47.5, −34.3)		<0.001
HbA_1c_ (%)	120	6.8 (6.2–8.3)	667	10.5 (9.0–11.9)	784	−3.7 (−4.3, −3.1)		<0.001
Fasting glucose (mmol/l)	52	5.3 (4.6–6.4)	697	7.2 (5.7–9.1)	746	−1.4 (−2.5, −0.3)		<0.05
Fasting C-peptide (nmol/l)								
Median (IQR)	89	0.21 (0.15–0.33)	681	0.10 (0–0.17)	767	0.19 (0.14, 0.24)		<0.001
≥0.2 nmol/l	89	52 (58.4)	681	128 (18.8)	767		12.7 (5.7, 28.3)	<0.001
>0.075 nmol/l	89	81 (91.0)	681	409 (60.1)	767		10.6 (4.3, 25.9)	<0.001
Insulin treatment	119	86 (72.3)	736	722 (98.1)	851		0.02 (0.004, 0.07)	<0.05
Ketonuria (moderate/large)	54	12 (22.2)	690	541 (78.4)	740		0.08 (0.04, 0.20)	<0.001
BMI-SDS	64	0.05 (−0.77 to 0.77)	731	−0.63 (−1.47 to 0.23)	791	0.61 (0.20, 1.02)		<0.01

^a^*p* value from regression analysis, adjusted for sex, having a first-degree relative with type 1 diabetes, age and calendar year at stage 3 diagnosis

Similar findings were obtained when comparing clinical outcome variables between Fr1da and DiMelli children who were diagnosed with stage 3 type 1 diabetes at a younger age (<6 years) and at an older age (≥6 years) (ESM Table 1). Similar findings were also obtained when restricting the analysis to children with fasting C-peptide levels ≥0.2 nmol/l (ESM Table 2) and in a complete-case multivariate regression analysis (ESM Table 3).

### Determinants of clinical presentation at diagnosis of stage 3 type 1 diabetes

Fourteen of the 128 children with a prior early-stage diagnosis from the Fr1da study did not participate in metabolic staging and education. As compared with the 114 children who did participate, these children had higher HbA_1c_ levels at the time of stage 3 type 1 diabetes diagnosis and more hospitalisation days (ESM Table 4).

In the Fr1da study, the clinical presentation at diagnosis of stage 3 type 1 diabetes was not different between children who had or did not have a first-degree relative with type 1 diabetes (ESM Table 5). In contrast, children of the DiMelli study who had a first-degree relative with type 1 diabetes had lower HbA_1c_ (median [IQR], 78 mmol/mol [67–89] vs 92 mmol/mol [77–108]; 9.3% [8.3–10.3] vs 10.6% [9.2–12.0]; *p*<0.001) and fasting glucose levels (median [IQR], 5.6 mmol/l [4.7–7.5] vs 7.2 mmol/l [5.8–9.2]; 100 mg/dl [84–135] vs 130 mg/dl [104–165]; *p*<0.001) and less frequent moderate or large ketonuria at diagnosis of stage 3 type 1 diabetes (51.2% vs 80.1%; *p*<0.001) than DiMelli children without a first-degree relative with type 1 diabetes, but HbA_1c_, fasting C-peptide and ketonuria did not reach levels or frequencies observed in the children with a first-degree relative with type 1 diabetes from the Fr1da study (ESM Fig. 2).

Since the COVID-19 pandemic is reported to affect diagnosis, we assessed whether the clinical presentation at diagnosis of stage 3 type 1 diabetes in children from the Fr1da study differed between children diagnosed before and during the COVID-19 pandemic. No difference in clinical presentation was observed between children who developed stage 3 type 1 diabetes from August 2015 to February 2020 and children who developed stage 3 type 1 diabetes from March 2020 to December 2022 (ESM Table 6).

## Discussion

This study found that children who had been previously diagnosed with presymptomatic type 1 diabetes in a public health screening programme had a milder clinical presentation at diagnosis of stage 3 type 1 diabetes than children without prior screening for islet autoantibodies. This included lower HbA_1c_ and fasting blood glucose levels, higher fasting C-peptide levels, fewer children with ketonuria, fewer children requiring insulin at diagnosis, a low prevalence of DKA and normal BMI-SDS, possibly reflecting less weight loss.

Strengths of our study are that participants with a prior early-stage diagnosis were not a priori selected on a genetic susceptibility criterion, but population-based, and that the comparison group was recruited from the same geographic region. Both cohorts were islet autoantibody-positive at diagnosis of stage 3 type 1 diabetes and were comparable in age, sex, GRS and frequency of HLA genotypes previously associated with type 1 diabetes risk. A limitation of this study was that the Fr1da screening was conducted between February 2015 and December 2022 and included the COVID-19 pandemic period, whereas the recruitment of children in DiMelli occurred between 2009 and 2018. However, no differences were observed in the clinical variables examined between children in Fr1da who developed stage 3 type 1 diabetes before or during the pandemic. It is possible that the DiMelli cohort includes a minority of children who had islet autoantibody screening performed without our knowledge. No information on socioeconomic status was available and, for some variables, the proportion of missing data was high. However, similar findings were observed when performing a complete-case analysis, suggesting that bias through missing data is limited. Nevertheless, confirmation of our results with a larger sample size is warranted.

The findings support the hypothesis that screening for presymptomatic early stages of type 1 diabetes reduces disease severity at clinical onset. Moreover, the observations are largely consistent with previous findings. The lower HbA_1c_ levels observed at clinical manifestation in the Fr1da study were comparable to those of children in The Environmental Determinants of Diabetes in the Young (TEDDY) observational study who underwent regular metabolic monitoring after seroconversion of multiple islet autoantibodies [31]. Furthermore, HbA_1c_ levels in the DiMelli comparison cohort were comparable to those of children followed in the Diabetes Prospective Follow-up Registry (DPV), which, similar to DiMelli, captures metabolic variables at the time of manifestation without prior screening [32]. The lower HbA_1c_ and fasting blood glucose and increased fasting C-peptide concentrations indicate that a timely diagnosis was made in Fr1da cases. We would attribute this to both the diagnosis that came from the islet autoantibody results and the education and follow-up programme in which families were informed and reminded of the symptoms associated with the onset of stage 3 type 1 diabetes, as well as being monitored for worsening of metabolic variables. The low prevalence of DKA observed irrespective of whether families participated in education and monitoring or were followed during the lockdown period in the COVID-19 pandemic suggests that notification of islet autoantibody status is sufficient to raise awareness of the disease among families and paediatricians. The slightly worse metabolic status of individuals who declined education and follow-up indicates that early-stage diagnosis should include follow-up and monitoring to achieve optimal benefit for patients; however, this finding was limited by the small sample size and needs further confirmation.

An important observation was the higher fasting C-peptide concentrations and reduced need for insulin therapy in the Fr1da cases, indicating a higher beta cell reserve. These findings are also consistent with those in the TEDDY study [31]. Preservation of beta cell function was associated with a lower risk of hypoglycaemia and microvascular complications in the DCCT [33]. A threshold of 0.075 nmol/l for fasting C-peptide levels is proposed to define preserved beta cell function [33]. More than 90% of children from the Fr1da cohort had a fasting C-peptide ≥0.075 nmol/l and 58.4% ≥0.2 nmol/l. While it is expected that C-peptide will decline, the substantially higher C-peptide at diagnosis of stage 3 type 1 diabetes will increase eligibility for clinical trials aimed at preserving residual beta cell function and likely improve the response to intervention drugs used in these trials [34].

An objective of the Fr1da study was to prevent or significantly reduce the incidence of DKA and hospitalisation in children diagnosed with stage 3 type 1 diabetes. An association of DKA at the onset of clinical diabetes with increased mortality, prolonged hospital stays and increased ICU admission rate [35], as well as with lower residual beta cell function, poorer metabolic control and higher insulin requirements until several years after diagnosis, is well established [14, 31, 36, 37]. In addition to the individual burden of short- and long-term health consequences, the occurrence of DKA is also associated with increased costs to the healthcare system. Consistent with these associations, we observed a shorter median length of hospitalisation among children in the Fr1da study compared with data from the DPV cohort [35], and a lower incidence of children admitted to the ICU. It is expected that length of hospitalisation can be further reduced as soon as more highly qualified outpatient services are established for paediatric patients with newly diagnosed stage 3 type 1 diabetes.

Overall, the clinical presentation of children participating in the population-based Fr1da screening was comparable to that of children diagnosed with presymptomatic type 1 diabetes by islet autoantibody screening in natural history studies [7, 11], or clinical trials [38], and similarly with respect to stress and anxiety levels in parents in response to screening. Previous findings from the Fr1da study indicated that psychological stress scores were transiently elevated in mothers of children diagnosed with presymptomatic type 1 diabetes in the Fr1da screening and that the distress reported by the families was low or moderate in the majority of families [18], consistent with findings in children diagnosed with islet autoantibodies in natural history studies [39, 40] and in parents of children identified with an increased genetic risk for type 1 diabetes [41, 42]. With an appropriate education and care programme, islet autoantibody screening and a diagnosis of presymptomatic type 1 diabetes appear unlikely to lead to the level of parental stress observed in families of children diagnosed with stage 3 type 1 diabetes without prior screening [18]. A young age at stage 3 type 1 diabetes diagnosis and no family history of type 1 diabetes are associated with a more severe clinical presentation of diabetes, including a higher frequency of DKA [11]. This was also observed in the DiMelli cohort. Children in the Fr1da cohort had a milder diabetes onset regardless of age. No differences in clinical outcome variables were observed between Fr1da children with and without a first-degree relative with type 1 diabetes. It is therefore unlikely that the higher proportion of children with a first-degree relative in the Fr1da study resulted in a better clinical presentation; however, this finding needs to be confirmed with a larger sample size. Furthermore, in contrast to several previous reports [10, 43], in the Fr1da study no differences in clinical presentation were observed between children diagnosed before and during the COVID-19 pandemic. Overall, our results suggest that children both with and without first-degree relatives with type 1 diabetes benefit from screening, education and metabolic monitoring, and that clinical benefit was not affected by the COVID-19 pandemic.

In conclusion, this study suggests clinical benefit of a public health screening for type 1 diabetes. By identifying children with presymptomatic type 1 diabetes and offering them participation in education and metabolic follow-up, the clinical presentation at manifestation of stage 3 type 1 diabetes is improved. Of particular note are the low prevalence of DKA, lower rates of hospitalisation and ICU admissions, and better-preserved residual beta cell function. These results may inform considerations of screening children for presymptomatic type 1 diabetes at a population-based level.

## Supplementary Information

Below is the link to the electronic supplementary material.

Supplementary file1 (PDF 302 KB)

## Data Availability

The de-identified individual participant data that underlie the results (text, tables, figures and ESM, excluding genetic data) reported in this article can be shared between 9 and 36 months after publication of the article. Requests will be honoured from researchers who provide a methodologically sound proposal and who complete a Data Use Agreement with Helmholtz Munich. Requests should be directed by email to the corresponding authors.

## Abbreviations

DKA: Diabetic ketoacidosis
DPV: Diabetes Prospective Follow-up Registry
GRS: Genetic risk score
ICU: Intensive care unit
SDS: SD score
TEDDY: The Environmental Determinants of Diabetes in the Young
