# Supplementary material for: Children diagnosed with presymptomatic type 1 diabetes through public health screening have milder diabetes at clinical manifestation
Source: Diabetologia. 2023 Jun 17;66(9):1633–42. doi: 10.1007/s00125-023-05953-0 (PMC10390633; doi:10.1007/s00125-023-05953-0)

Electronic Supplemental Material

Hummel et al: Children diagnosed with presymptomatic type 1 diabetes through public health screening have milder diabetes at clinical manifestation

Table of contents:

- ESM Table 1. Clinical presentation at diagnosis of stage 3 type 1 diabetes relative to age at diagnosis in children previously diagnosed with presymptomatic type 1 diabetes in the Fr1da study compared with children diagnosed with incident type 1 diabetes in the DiMelli study who had not participated in prior screening.
- ESM Table 2. Clinical presentation at diagnosis of stage 3 type 1 diabetes in children with C-peptide levels ≥0.2 nmol/l at stage 3 diagnosis who were previously diagnosed with presymptomatic type 1 diabetes in the Fr1da study compared with children diagnosed with incident type 1 diabetes in the DiMelli study without prior screening.
- ESM Table 3. Complete case multivariate regression analysis on the effect of diagnosis of presymptomatic diabetes in the Fr1da study (N=15) on clinical presentation at diagnosis of stage 3 type 1 diabetes compared to children diagnosed with incident type 1 diabetes in the DiMelli study (N=572) without prior screening.
- ESM Table 4. Clinical presentation at diagnosis of stage 3 type 1 diabetes relative to participation in metabolic staging and education in children previously diagnosed with presymptomatic type 1 diabetes in the Fr1da study.
- ESM Table 5. Clinical presentation at diagnosis of stage 3 type 1 diabetes relative to family history of type 1 diabetes in children previously diagnosed with presymptomatic type 1 diabetes in the Fr1da study compared with children diagnosed with incident type 1 diabetes in the DiMelli study who had not participated in prior screening.
- ESM Table 6. Clinical presentation at diagnosis of stage 3 type 1 diabetes before versus during the COVID-19 pandemic in children previously diagnosed with presymptomatic type 1 diabetes in the Fr1da study.
- ESM Figure 1. Fr1da study participants
- ESM Figure 2. HbA_{1c}, fasting glucose, fasting C-peptide and BMI-SDS at diagnosis of stage 3 type 1 diabetes are shown for children with a first-degree relative with type 1 diabetes previously diagnosed with presymptomatic type 1 diabetes in the Fr1da study

compared with children with a first-degree relative with type 1 diabetes diagnosed with incident type 1 diabetes in the DiMelli study.

ESM Table 1 Clinical presentation at diagnosis of stage 3 type 1 diabetes relative to age at diagnosis in children previously diagnosed with presymptomatic type 1 diabetes in the Fr1da study compared with children diagnosed with incident type 1 diabetes in the DiMelli study who had not participated in prior screening.

	Fr1da		DiMelli		p-value
	n	Median (IQR) or n (%)	n	Median (IQR) or n (%)	
**Age at stage 3 T1D diagnosis < 6 years**					
Age at diagnosis (years)	51	4.7 (3.3-5.3)	281	3.8 (2.3-5.0)	<0.01
Sex	51		281		0.8
Female		25 (49.0)		145 (51.6)	
Male		26 (51.0)		136 (48.4)	
Clinical parameters at diagnosis					
HbA_{1c} (mmol/mol)	49	49 (45-60)	245	84 (69-97)	<0.001*
HbA_{1c} (%)	49	6.6 (6.3-7.6)	245	9.8 (8.5-11.0)	<0.001*
Fasting glucose (mmol/l)	23	5.1 (4.4-5.9)	261	6.5 (5.0-8.5)	0.7*
Fasting C-Peptide (nmol/l)	38	0.18 (0.14-0.26)	253	0.07 (0-0.13)	<0.001*
Insulin treatment	49	35 (71.4)	281	278 (98.9)	<0.001*
Ketonuria (moderate/large)	24	3 (12.5)	263	197 (74.9)	<0.001*
DKA	50	1 (2.0)			
pH	32	7.40 (7.39-7.42)			
Symptoms	49	24 (49.0)			
Hospitalisation	49	39 (79.6)			
Days	47	9 (4-12)			
Intensive care unit (yes)	47	1 (2.1)			
**Age at stage 3 T1D diagnosis ≥ 6 years**					
Age at diagnosis (years)	77	8.5 (7.0-10.3)	455	8.8 (7.5-10.0)	0.6
Sex	77		455		0.6
Female		34 (44.2)		217 (47.7)	
Male		43 (55.8)		238 (52.3)	
Clinical parameters at diagnosis					
HbA_{1c} (mmol/mol)	71	51 (44-73)	422	95 (80-112)	<0.001*
HbA_{1c} (%)	71	6.8 (6.2-8.8)	422	10.8 (9.5-12.4)	<0.001*
Fasting glucose (mmol/l)	29	5.4 (5.1-6.4)	436	7.5 (6.1-9.5)	<0.05*
Fasting C-Peptide (nmol/l)	51	0.25 (0.16-0.37)	428	0.10 (0.07-0.17)	<0.001*
Insulin treatment	70	51 (72.9)	455	444 (97.6)	<0.001*
Ketonuria (moderate/large)	30	9 (30.0)	427	344 (80.6)	<0.001*
DKA	68	2 (2.9)			
pH	36	7.40 (7.37-7.42)			
Symptoms	72	29 (40.3)			
Hospitalisation	72	51 (70.8)			
Days	70	8 (0-10)			
Intensive care unit (yes)	61	1 (1.6)			

*adjusted for sex, having a first-degree relative with type 1 diabetes, age at and calendar year at stage 3 diagnosis; T1D: type 1 diabetes, DKA: diabetic ketoacidosis

ESM Table 2 Clinical presentation at diagnosis of stage 3 type 1 diabetes in children with C-peptide levels ≥0.2 nmol/l at stage 3 diagnosis who were previously diagnosed with presymptomatic type 1 diabetes in the Fr1da study compared with children diagnosed with incident type 1 diabetes in the DiMelli study without prior screening.

	Fr1da children with C-peptide ≥0.2 nmol/l at stage 3 diagnosis		DiMelli children with C-peptide ≥0.2 nmol/l at stage 3 diagnosis		Adjusted p-value*
	n	Median (IQR) or n (%)	n	Median (IQR) or n (%)	
HbA_{1c} (mmol/mol)	51	46 (41; 54)	121	80 (63; 96)	<0.001
HbA_{1c} (%)	51	6.4 (5.9; 7.1)	121	9.5 (8.0; 10.9)	<0.001
Fasting glucose (mmol/l)	27	5.4 (5.1; 6.3)	119	7.1 (5.8; 8.9)	0.1
Fasting C-peptide (nmol/l)	52	0.30 (0.22;0.44)	128	0.26 (0.20; 0.33)	0.1
Insulin treatment	49	26 (53.1)	128	121 (94.5)	<0.05
Ketonuria (moderate/large)	19	1 (5.3)	115	75 (65.2)	<0.05
BMI-SDS	29	0.12 (-0.50; 1.74)	127	0.02 (-0.85;0.93)	0.2

***** adjusted for sex, age and calendar year at stage 3 type 1 diabetes diagnosis, having a first-degree relative with type 1 diabetes

ESM Table 3 Complete case multivariate regression analysis on the effect of diagnosis of presymptomatic diabetes in the Fr1da study (N=15) on clinical presentation at diagnosis of stage 3 type 1 diabetes compared to children diagnosed with incident type 1 diabetes in the DiMelli study (N=572) without prior screening.

	Fr1da<br>Median (IQR) or n (%)	DiMelli<br>Median (IQR) or n (%)	ß – Estimate<br>(95% CI)	Odds Ratio<br>(95% CI)	Adjusted<br>p-value*
HbA_{1c} (mmol/mol)	51 (39; 63)	92 (77; 108)	-41 (-53; -30)		<0.001
HbA_{1c} (%)	6.8 (5.7; 7.9)	10.6 (9.2; 12.0)	-3.8 (-4.9; -2.7)		<0.001
Fasting glucose (mmol/l)	5.3 (4.8; 7.5)	7.2 (5.8-9.1)	-0.2 (-1.8; 1.5)		0.8
Fasting C-Peptide (nmol/l)					
Median (IQR)	0.18 (0.13; 0.21)	0.10 (0; 0.17)	0.12 (0.05; 0.18)		<0.001
≥0.2 nmol/l	7 (46.7)	100 (17.5)		12.0 (3.4; 42.0)	<0.001
>0.075 nmol/l	13 (86.7)	341 (59.6)		9.3 (1.9; 45.1)	<0.01
Insulin treatment	11 (73.3)	564 (98.6)		0.02 (0.002; 0.17)	<0.001
Ketonuria (moderate/large)	2 (13.3)	445 (77.8)		0.05 (0.01; 0.25)	<0.001
BMI-SDS	0.20 (-0.56; 0.75)	-0.62 (-1.43; 0.23)	0.90 (0.18; 1.62)		<0.05

*****Adjusted for sex, having a first-degree relative with type 1 diabetes, age and calendar year at stage 3 type 1 diabetes diagnosis

ESM Table 4 Clinical presentation at diagnosis of stage 3 type 1 diabetes relative to participation in metabolic staging and education in children previously diagnosed with presymptomatic type 1 diabetes in the Fr1da study.

	Children underwent metabolic staging and education		Metabolic staging and education pending/declined		
	n	Median (IQR) or n (%)	n	Median (IQR) or n (%)	p-value
Age at diagnosis of stage 3 T1D (years)	114	6.7 (5.0-9.3)	14	6.7 (4.9-7.7)	0.3
Sex	114		14		0.6
Female		54 (47.4)		5 (35.7)	
Male		60 (52.6)		9 (64.3)	
**Clinical parameters at diagnosis of stage 3 T1D**					
HbA_{1c} (mmol/mol)	110	51 (44-63)	10	72 (52-92)	<0.01
HbA_{1c} (%)	110	6.8 (6.2-7.9)	10	8.7 (6.9-10.6)	<0.01
Insulin treatment	108	75 (69.4)	11	11 (100)	0.1
DKA	107	2 (1.9)	11	1 (9.1)	0.3
Symptoms	108	42 (38.9)	13	11 (84.6)	<0.01
Hospitalisation					
Days	106	8 (0-10)	11	11 (9-15)	<0.01
Intensive care unit (yes)	97	2 (2.1)	11	0	0.2

T1D: type 1 diabetes, DKA: diabetic ketoacidosis

ESM Table 5 Clinical presentation at diagnosis of stage 3 type 1 diabetes relative to family history of type 1 diabetes in children previously diagnosed with presymptomatic type 1 diabetes in the Fr1da study compared with children diagnosed with incident type 1 diabetes in the DiMelli study who had not participated in prior screening.

	Fr1da Children with first-degree relative with T1D		Fr1da Children without first-degree relative with T1D			DiMelli Children with first-degree relative with T1D		DiMelli Children without first-degree relative with T1D		
	n	Median (IQR) or n (%)	n	Median (IQR) or n (%)	p-value	n	Median (IQR) or n (%)	n	Median (IQR) or n (%)	p-value
Age at diagnosis of stage 3 T1D (years)	18	8.3 (4.8-11.2)	110	6.7 (5.0-8.7)	0.2	49	6.5 (3.9-9.4)	683	7.3 (4.5-9.2)	0.5
Sex	18		110		0.2	49		683		0.6
Female		6 (33.3)		53 (48.2)			22 (44.9)		338 (49.5)	
Male		12 (66.7)		57 (51.8)			27 (55.1)		345 (50.5)	
**Clinical parameters at diagnosis of stage 3 T1D**										
HbA_{1c} (mmol/mol)	18	50 (43-65)	102	51 (45-67)	0.6	44	78 (67-89)	620	92 (77-108)	<0.001
HbA_{1c} (%)	18	6.7 (6.1-8.1)	102	6.8 (6.3-8.3)	0.6	44	9.3 (8.3-10.3)	620	10.6 (9.2-12.0)	<0.001
Fasting glucose (mmol/l)	7	5.4 (4.8-6.9)	45	5.3 (4.6-6.3)	0.5	49	5.6 (4.7-7.5)	645	7.2 (5.8-9.2)	<0.001
Fasting C-Peptide (nmol/l)	12	0.22 (0.16-0.56)	77	0.21 (0.13-0.31)	0.3	48	0.10 (0.0-0.17)	630	0.10 (0.0-0.17)	0.6
Insulin treatment	18	12 (66.7)	101	74 (73.3)	0.6	49	47 (95.9)	683	671 (98.2)	0.2
Ketonuria (moderate/large)	8	2 (25.0)	46	10 (21.7)	0.8	43	22 (51.2)	643	515 (80.1)	<0.001
DKA	18	0 (0)	100	3 (3.0)	0.5					
pH	10	7.39 (7.38-7.42)	58	7.40 (7.38-7.42)	0.9					
Symptoms	18	8 (44.4)	103	45 (43.7)	1.0					
Hospitalisation	18	13 (72.2)	103	77 (74.8)	0.8					
Days	17	7 (0-8.5)	100	9 (0-11.8)	0.1					
Intensive care unit (yes)	17	0 (0)	91	2 (2.2)	0.4					

T1D: type 1 diabetes, DKA: diabetic ketoacidosis

ESM Table 6 Clinical presentation at diagnosis of stage 3 type 1 diabetes before versus during the COVID-19 pandemic in children previously diagnosed with presymptomatic type 1 diabetes in the Fr1da study.

	Stage 3 Diagnosis 8/2015 -2/2020		Stage 3 Diagnosis 3/2020 – 11/2022		p-value
	n	Median (IQR) or n (%)	n	Median (IQR) or n (%)	
Age at diagnosis of stage 3 T1D (years)	56	5.5 (3.9-7.3)	72	8.0 (5.9-10.2)	<0.001
Sex	56		72		0.3
Female		29 (51.8)		30 (41.7)	
Male		27 (48.2)		42 (58.3)	
**Clinical parameters at diagnosis of stage 3 T1D**					
HbA_{1c} (mmol/mol)	52	48 (46-65)	68	52 (44-69)	0.4
HbA_{1c} (%)	52	6.6 (6.3-8.1)	68	6.9 (6.2-8.5)	0.4
Fasting glucose (mmol/l)	23	5.1 (4.4-5.7)	29	5.5 (4.9-6.6)	0.09
Fasting C-Peptide (nmol/l)	42	0.20 (0.16-0.31)	47	0.22 (0.11-0.35)	0.8
Insulin treatment	51	37 (72.5)	68	49 (72.1)	0.7
Ketonuria (moderate/large)	29	4 (13.8)	25	8 (32.0)	0.1
DKA	52	2 (3.8)	66	1 (1.5)	0.4
pH	31	7.40 (7.37-7.42)	37	7.40 (7.38-7.42)	0.8
Symptoms	52	24 (46.2)	69	29 (42.0)	0.7
Hospitalisation	52	39 (75.0)	69	51 (73.9)	0.9
Days	51	9 (0-12)	66	8 (0-10)	0.2
Intensive care unit (yes)	49	1 (2.0)	59	1 (1.7)	0.8

T1D: type 1 diabetes, DKA: diabetic ketoacidosis

ESM Figure 1 Fr1da study participants

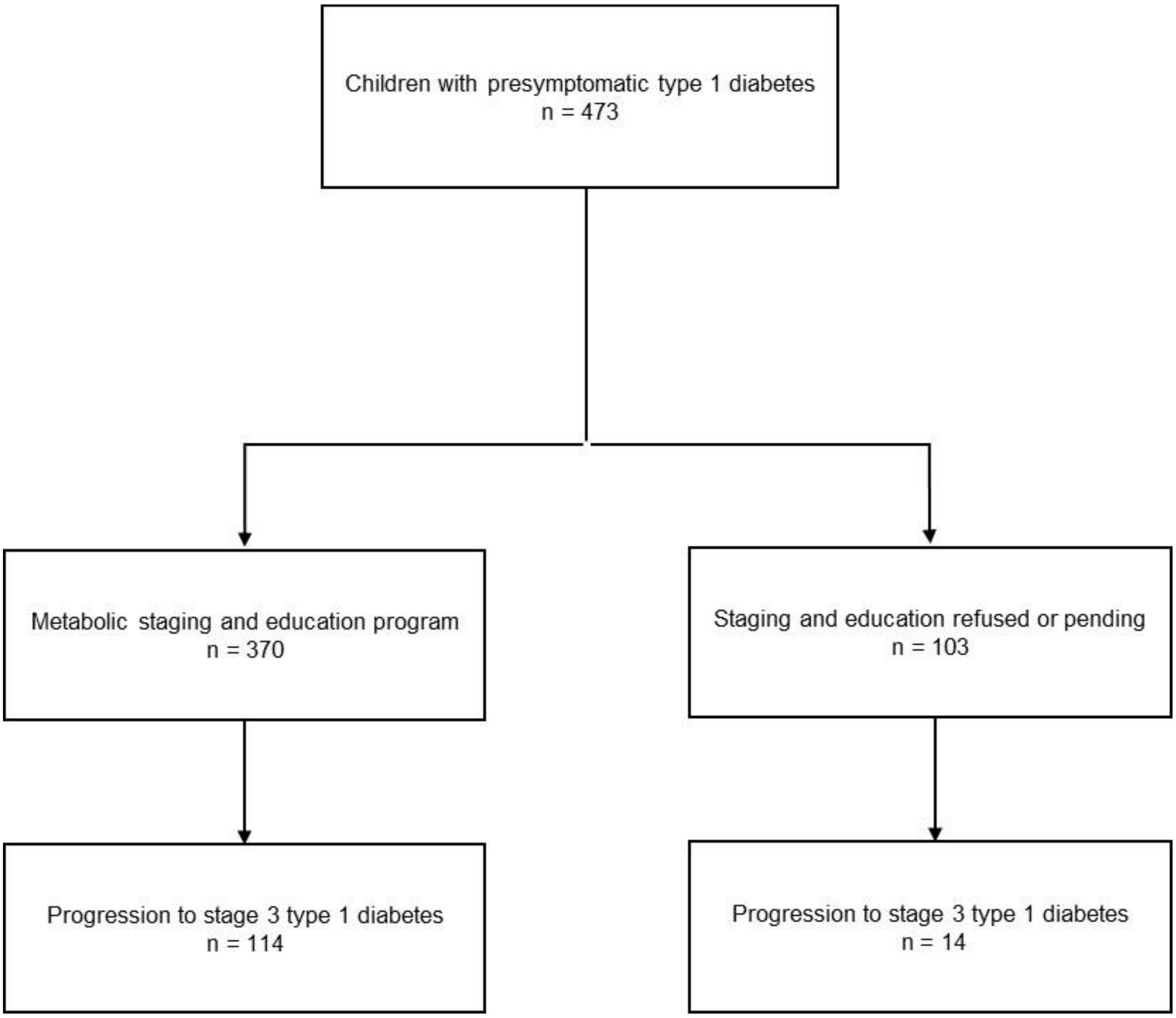

ESM Figure 2 HbA_{1c}, fasting glucose, fasting C-peptide and BMI-SDS at diagnosis of stage 3 type 1 diabetes are shown for children with a first-degree relative with type 1 diabetes previously diagnosed with presymptomatic type 1 diabetes in the Fr1da study compared with children with a first-degree relative with type 1 diabetes diagnosed with incident type 1 diabetes in the DiMelli study.

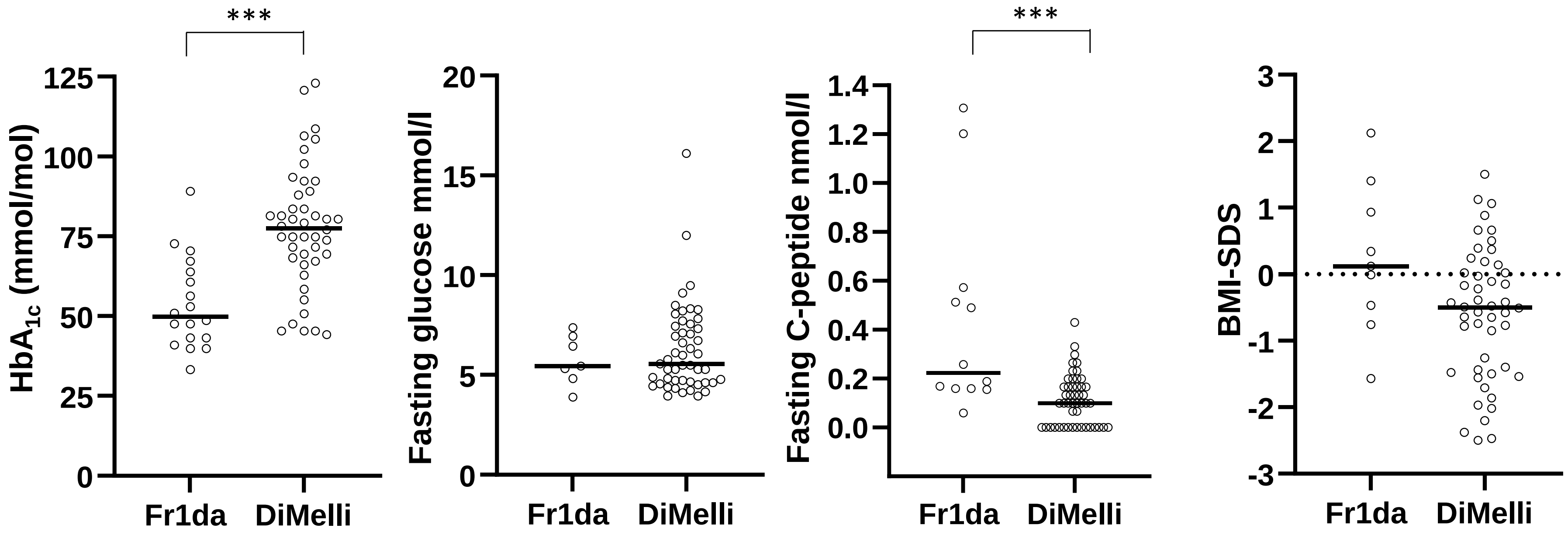

Supplement: Supplementary file 1 — Supplementary file1 (PDF 302 KB) [file 125_2023_5953_MOESM1_ESM.pdf]
